# Environment-Dependent Downlink Pinching-Antenna Systems: Spectral–Energy Efficiency Tradeoffs and Design

**DOI:** 10.3390/s26072051

**Published:** 2026-03-25

**Authors:** Xiangyu Zha, Yongji Chen, Qi Wang

**Affiliations:** 1 School of Applied Mathematics, Nanjing University of Information Science and Technology, Nanjing 210044, China; xiangyuzha@nuist.edu.cn; 2 School of Applied Mathematics, University of Reading, Reading RG6 6DX, UK; 3 School of Data Science and Big Data Technology, Nanjing University of Information Science and Technology, Nanjing 210044, China; yongjichen@nuist.edu.cn; 4 School of Data Science and Big Data Technology, University of Reading, Reading RG6 6DX, UK; 5 School of Computer Science, Nanjing University of Information Science and Technology, Nanjing 210044, China

**Keywords:** pinching-antenna systems, environment-division multiple access, spectral–energy efficiency

## Abstract

Pinching-antenna systems (PASSs) offer a low-complexity and reconfigurable solution for near-field downlink communications by deploying multiple radiating elements along a single waveguide. Existing studies mainly assume simplified propagation conditions or focus on spectral efficiency, while the impact of environment-dependent interference patterns arising from user-specific blockage conditions on energy-efficient design remains unclear. An energy-efficient downlink design for single-waveguide PASS based on environment-division multiple access (EDMA) is investigated. Under a given propagation environment, EDMA exploits user-dependent blockage and visibility differences through proper pinching-antenna placement, thereby inducing different multi-user interference patterns without increasing radio-frequency hardware complexity. We examine how such blockage-dependent interference influences the relationship between spectral efficiency and energy efficiency, and develop an energy-aware EDMA framework that jointly considers pinching-antenna locations and transmit power allocation under quality-of-service constraints. The resulting coupled design problem is solved through an alternating optimization procedure. EDMA is compared with conventional time-division multiple access (TDMA) using a unified hardware and power-consumption model. Numerical results reveal clear energy-efficiency threshold behaviors with respect to blockage intensity, user population, and service requirements. The results further show that EDMA can significantly outperform TDMA in specific operating regimes.

## 1. Introduction

In near-field wireless communications and high-frequency indoor coverage scenarios, base stations (BSs) are often constrained by the number of radio-frequency chains, power budgets, and hardware complexity [1,2]. At the same time, they are required to provide reliable downlink services to multiple users. As a result, enabling efficient multi-user access under limited wireless resources has become a fundamental problem in communication system design. Multiple access mechanisms determine how users share the available resources [3]. Their design directly affects both spectral efficiency and overall energy consumption. In this context, the tradeoff between multi-user interference suppression and resource utilization efficiency becomes a central challenge in downlink multi-user transmission.

To address this challenge, existing multiple access techniques mainly rely on exploiting separability across different resource domains. Time-division multiple access (TDMA) [4], frequency-division multiple access (FDMA) [5], and orthogonal frequency-division multiple access (OFDMA) allocate orthogonal resources in the time or frequency domain, which effectively avoids multi-user interference [6,7]. However, each user can only occupy a portion of the total bandwidth, which limits spectral efficiency. Code-division multiple access (CDMA) allows multiple users to access the entire bandwidth through code-domain separation [8], but typically requires higher receiver complexity. Furthermore, space-division multiple access (SDMA) and non-orthogonal multiple access (NOMA) enable concurrent transmission by exploiting spatial or power-domain differences [9,10]. These schemes often rely on accurate channel state information, sophisticated signal processing, and additional energy consumption. While such approaches have shown strong performance in conventional far-field systems or hardware-rich settings, their energy cost becomes increasingly pronounced in near-field communications with low-complexity architectures. These limitations make low-complexity and energy-aware multi-user access particularly important in near-field systems.

Pinching-antenna systems (PASSs) provide a structurally simple and energy-efficient solution for near-field downlink communications by flexibly deploying multiple reconfigurable radiating elements on a shared feeding structure [11,12]. Thanks to the decoupled waveguide-based feeding and radiation architecture, PASS enables flexible control of spatial radiation patterns without significantly increasing the number of radio-frequency chains, thereby introducing additional spatial degrees of freedom for multi-user transmission [13]. However, adjustable radiation alone does not eliminate multi-user interference. In downlink scenarios, user interference remains unavoidable and is jointly shaped by radiation geometry and the propagation environment, which fundamentally limits both spectral efficiency and energy efficiency. To address these challenges, existing studies have explored PASS from different perspectives. For instance, Ref. [14] maximizes the downlink rate by optimizing pinching-antenna locations while accounting for path loss and phase alignment, and proposes a low-complexity solution with near-optimal performance. In [15], PASS is extended to a downlink NOMA setting, where antenna locations and power allocation are jointly considered under QoS constraints, and closed-form optimal solutions are derived for simplified cases. In addition, Ref. [16] investigates downlink power allocation for NOMA-based PASS and develops a transmit-power-minimization framework subject to users’ individual service constraints, where superposition coding and successive interference cancellation are employed to support simultaneous multi-user transmission. Furthermore, Ref. [17] studies PA-assisted NOMA design by jointly considering antenna placement and fairness-oriented power allocation, and further employs a convolutional neural network to infer near-optimal power coefficients with reduced computational complexity. Moreover, Ref. [18] addresses the strong frequency selectivity and resulting inter-symbol interference in multi-user PASS by modeling the channel as a finite impulse response (FIR) filter and proposing an OFDMA-based resource allocation scheme that improves the minimum user rate with low computational complexity while ensuring fairness.

Beyond conventional signal-domain multiple access, PASS also provides a new degree of freedom for interference management through spatially reconfigurable radiating locations. In particular, because the radiating elements can be deployed at different positions along the waveguide, different pinching-antenna placements may lead to different user visibility and blockage relationships under a given propagation environment. This location-dependent propagation structure creates the possibility of distinguishing users not only through time, frequency, code, or power resources, but also through environment-dependent link conditions. Furthermore, Ref. [19] develops a power-radiation model for PASS and studies the corresponding optimal beamforming design. Against this background, EDMA has recently been proposed as a new multiple access paradigm [20]. Unlike traditional schemes that rely on orthogonalization in the time, frequency, or power domains, EDMA is built upon reconfigurable antenna architectures such as PASS. Rather than controlling the propagation environment itself, EDMA utilizes the user-dependent blockage and visibility differences associated with different pinching-antenna placements under a given environment. In this way, different PA locations give rise to different multi-user interference patterns in the environment domain. As a result, interference management is no longer solely handled by complex baseband signal processing, but is partially supported by the physical propagation structure, leading to a multiple access mechanism that is highly compatible with pinching-antenna systems.

Although EDMA introduces a new design dimension for multi-user communications, existing studies mainly focus on its potential benefits in terms of spectral efficiency or system throughput [21,22]. However, its role in energy-efficient system design remains insufficiently understood, especially when the interference pattern depends on user-specific blockage conditions and PA placement. Meanwhile, prior energy-efficiency studies on PASS typically assume simplified or fixed interference structures and do not account for the location-dependent interference variation enabled by EDMA. Therefore, under environment-driven multiple access built upon PASS, how the tradeoff between spectral efficiency and energy efficiency evolves, and under what system and environmental conditions the introduction of EDMA is energy-efficient and practically beneficial, remain open research questions. Based on the above observations and problem formulation, the main contributions of this paper are summarized as follows.

- This paper studies how blockage-dependent, environment-dependent interference patterns affect the spectral efficiency–energy efficiency (SE–EE) tradeoff in single-waveguide PASS. Under a given propagation environment, different pinching-antenna placements produce different user visibility and interference relationships, leading to SE–EE behaviors that differ from conventional time-division-based designs.
- An energy-aware downlink design based on EDMA is proposed. Under quality-of-service constraints, pinching-antenna locations and transmit power are jointly considered in a coupled design framework, capturing the tradeoff between desired-link enhancement and blockage-assisted interference mitigation, without increasing radio-frequency hardware complexity. The resulting problem is addressed through an alternating optimization procedure, where the two variable blocks are updated sequentially rather than simultaneously.
- Under a unified single-waveguide hardware and power model, EDMA is compared with conventional TDMA. The results identify energy-efficiency thresholds and operating regimes with respect to blockage intensity, user density, and service requirements, clarifying when environment-dependent access enabled by PA placement is more energy-efficient than time-domain orthogonalization.

## 2. System Model

**Figure 1:**
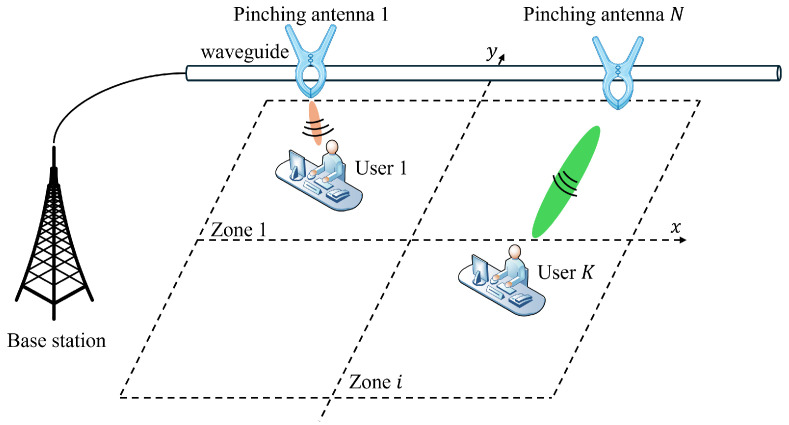
Architecture of a pinching-antenna-assisted downlink indoor NFC system.

As shown in Figure 1, a downlink multi-user communication scenario is considered, where a base station (BS) serves *K* single-antenna users through a single dielectric waveguide. The user set is denoted by K={1,2,…,K}. All users are located on the same horizontal plane and are randomly distributed within a square service area of size D×D centered at the origin. The dielectric waveguide is deployed horizontally at a height *d* above the ground. To better reflect practical deployment constraints, we assume that only a finite set of feasible pinching-antenna (PA) installation points is available along the waveguide. A set of candidate PA locations is predefined along the waveguide and can be selectively activated to form radiating apertures. Let *N* denote the total number of candidate PA locations, indexed by N={1,2,…,N}. The position of the *n*-th candidate PA is given by $\psi_{n}={\left[x_{n},\;0,\;d\right]}^{T}$, where $x_{n}$ denotes the ordered candidate PA locations along the waveguide within the service region. Accordingly, the PA deployment problem considered in this work is a discrete selection problem over the predefined candidate set rather than a fully continuous placement problem. Hence, the proposed design does not assume arbitrary relocation of antennas after deployment. Instead, it optimizes which candidate PAs are activated and assigned to different users under a given physical environment. The position of user *k* is denoted by $u_{k}={\left[x_{k},\;y_{k},\;0\right]}^{T}$. All pinching antennas are fed by the same dielectric waveguide, with the induced phase modeled as

(1) $$g_{n}=e^{-j\frac{2\pi }{\lambda_{g}}r_{n}},$$

where $r_{n}$ denotes the effective propagation distance along the waveguide from the feeding point to the *n*-th candidate PA, and $\lambda_{g}$ is the guided wavelength.

Each activated PA acts as an effective radiating point in space. The distance between the *n*-th PA and user *k* is given by

(2) $$r_{k,n}=∥u_{k}-\psi_{n}∥.$$

The corresponding free-space propagation coefficient is modeled as

(3) $$f_{k,n}=\frac{\sqrt{\eta }}{r_{k,n}}e^{-j\frac{2\pi }{\lambda }r_{k,n}},$$

where λ denotes the carrier wavelength and η is a path-loss-related constant. More specifically, η is introduced as a channel-side scaling factor that captures the distance-dependent free-space attenuation together with the effective radiation gain of the pinching antennas. It is used only in the propagation model to characterize the strength of the radiated PA–user link, and does not represent the electrical power injected into the waveguide or the hardware power-consumption efficiency.

By combining waveguide propagation and spatial radiation, the effective downlink channel coefficient between the *n*-th PA and user *k* is expressed as

(4) $$h_{k,n}=f_{k,n}g_{n}=\frac{\sqrt{\eta }}{r_{k,n}}e^{-j\frac{2\pi }{\lambda_{g}}r_{n}}e^{-j\frac{2\pi }{\lambda }r_{k,n}}.$$

Different from the idealized binary blockage treatment, we consider a more general visibility description to characterize the uncertainty caused by environmental blockages. In practical indoor near-field communication (NFC) scenarios, the visibility of each PA–user link depends on the local geometry, obstacle density, and PA–user separation, which provides a more realistic abstraction for random blockage effects. It should be emphasized that the visibility condition of each PA–user link is determined by the propagation environment and the PA–user geometry, rather than by the optimization algorithm itself. The role of PA activation is only to select, from the predefined candidate set, which PA–user links are utilized for transmission under the given environment-dependent visibility conditions. Therefore, the proposed design does not control the propagation environment itself; instead, it exploits the differences in environment-dependent visibility associated with different candidate PA locations under a given physical environment.

Moreover, to improve the realism of the energy-efficiency analysis, the total power consumption is modeled by accounting for multiple hardware-related components, including the radiated transmit power, the RF-chain power, the PA-control power, and the circuit power. Hence, the total power consumption is expressed as

(5) $$P_{\mathrm{tot}}=\frac{1}{\xi_{\mathrm{pa}}}P_{\mathrm{tx}}+P_{\mathrm{RF}}+N_{\mathrm{act}}P_{\mathrm{PA}}+P_{\mathrm{sta}},$$

where $P_{\mathrm{tx}}=\sum_{i=1}^{K}P_{i}$ denotes the total radiated transmit power over all user data streams, and $P_{i}$ is the transmit power allocated to the symbol intended for user *i*. Here, $\xi_{\mathrm{pa}}$ denotes the power-amplifier efficiency, $P_{\mathrm{RF}}$ is the circuit power consumed by the single RF chain, $N_{\mathrm{act}}$ is the number of activated PAs, $P_{\mathrm{PA}}$ denotes the control and activation power consumed per active PA, and $P_{\mathrm{sta}}$ represents the residual static hardware power, such as baseband processing and auxiliary circuitry. This refined power model enables a more faithful characterization of the practical energy cost of PASS deployment.

### 2.1. Downlink Transmission Model for PASS

Let $s_{i}$ denote the baseband symbol intended for user *i*, with $E\{|s_{i}{|}^{2}\}=1$. Denote by $A_{i}\subseteq N$ the set of activated pinching antennas serving user *i*. The effective downlink channel from data stream *i* to user *k* is defined as

(6) $$H_{k,i}≜\sum_{n\in A_{i}}{\tilde{h}}_{k,n},$$

where ${\tilde{h}}_{k,n}$ denotes the effective PA–user channel coefficient after incorporating the environment-dependent visibility or blockage condition associated with the link between the *n*-th PA and user *k*. Hence, $H_{k,i}$ aggregates the contributions of all activated pinching antennas assigned to stream *i*. The effective channel $H_{k,i}$ captures both the deterministic waveguide-induced phase and the distance-dependent near-field radiation effects through ${\tilde{h}}_{k,n}$. Accordingly, the received signal at user *k* is expressed as

(7) $$y_{k}=\sum_{i=1}^{K}\sqrt{P_{i}}\;H_{k,i}\;s_{i}+n_{k},$$

where $n_{k}∼\mathrm{CN}\left(0,\sigma^{2}\right)$ denotes additive white Gaussian noise (AWGN). Moreover, the interference experienced by user *k* is determined by the cross-link effective channels ${\left\{H_{k,i}\right\}}_{i\neq k}$, whose values depend on the activated PA sets and the corresponding environment-dependent visibility conditions, rather than on any direct control of the propagation environment itself.

#### 2.1.1. TDMA as a Baseline Scheme

Under TDMA, only one user is scheduled in each time slot. As a result, $H_{k,i}=0$ for all i≠k, and the received signal at user *k* reduces to

(8) $$y_{k}=\sqrt{P_{k}}H_{k,k}s_{k}+n_{k}.$$

Assuming equal time allocation among the *K* users to ensure fairness, the achievable downlink rate of user *k* is given by

(9) $$R_{k}^{\mathrm{TDMA}}=\frac{1}{K}\log_{2}\;\left(1+\frac{P_{k}{\left|H_{k,k}\right|}^{2}}{\sigma^{2}}\right).$$

Since no inter-user interference is present under TDMA, the quantity inside the logarithm is the received signal-to-noise ratio (SNR). More generally, if user *k* is assigned a time fraction $\tau_{k}$ with $\sum_{k=1}^{K}\tau_{k}=1$, the corresponding TDMA rate can be written as

(10) $$R_{k}^{\mathrm{TDMA}}=\tau_{k}\log_{2}\;\left(1+\frac{P_{k}{\left|H_{k,k}\right|}^{2}}{\sigma^{2}}\right),$$

where the equal-time benchmark considered in this paper corresponds to $\tau_{k}=1/K$ for all users. Therefore, TDMA eliminates inter-user interference through time-domain orthogonalization, but at the cost of reducing the effective transmission time available to each user. This tradeoff makes TDMA a meaningful baseline for evaluating whether EDMA can improve the SE–EE relationship under the same hardware setting.

#### 2.1.2. EDMA with Environment-Dependent Link Visibility and Interference

To characterize environment-dependent link visibility in a more realistic manner, the effective PA–user link is modeled as

(11) $${\tilde{h}}_{k,n}≜b_{k,n}h_{k,n},$$

where $h_{k,n}$ denotes the cascaded PA–user channel coefficient defined above, $b_{k,n}$ is a Bernoulli random variable, and $\rho_{k,n}\in \left[0,1\right]$ denotes the visibility probability of the link between the *n*-th PA and user *k*, i.e., the probability that this PA–user path remains unblocked under the given propagation environment.

(12) $$b_{k,n}∼\mathrm{Bernoulli}\left(\rho_{k,n}\right),$$

with $\mathrm{Pr}(b_{k,n}=1)=\rho_{k,n}$ and $\mathrm{Pr}(b_{k,n}=0)=1-\rho_{k,n}$. Here, $b_{k,n}=1$ indicates that the link is visible, while $b_{k,n}=0$ means that the propagation path is blocked by the environment. Compared with the binary deterministic visibility indicator, this formulation captures the stochastic nature of blockage and provides a more practically meaningful abstraction for EDMA-enabled PASS systems.

Under this probabilistic visibility model, the environment determines the statistical existence of each candidate PA–user link, while PA activation and assignment determine which of these candidate links are effectively exploited for transmission. Therefore, EDMA does not change the physical blockage condition itself. Instead, it leverages the given environment-dependent visibility structure to alter the effective link-utilization pattern and, consequently, the expected multi-user interference topology in a statistical sense. Meanwhile, blockage may suppress not only interference links but also desired links. Therefore, EDMA design must balance interference mitigation and desired-link reliability, rather than relying on blockage alone. This point is particularly important for the present problem, since the energy-efficiency benefit of EDMA does not arise from any direct manipulation of the environment, but from selecting and allocating candidate PAs in a way that better matches the given statistical visibility pattern.

Under EDMA, multiple users are served concurrently within the same time slot, and inter-user interference naturally arises. Based on the unified signal model in (7) together with the environment-dependent link definition in (11), the received signal at user *k* consists of the desired signal component, multi-user interference, and AWGN.

Accordingly, for a given realization of the blockage variables $b_{k,n}$, the standard signal-to-interference-plus-noise ratio (SINR) of user *k* under EDMA is given by

(13) $${\mathrm{SINR}}_{k}^{\mathrm{EDMA}}=\frac{P_{k}{\left|H_{k,k}\right|}^{2}}{\sum_{i\neq k}P_{i}{\left|H_{k,i}\right|}^{2}+\sigma^{2}},$$

and the achievable downlink rate is expressed as

(14) $$R_{k}^{\mathrm{EDMA}}=\log_{2}\;\left(1+{\mathrm{SINR}}_{k}^{\mathrm{EDMA}}\right).$$

For analytical tractability, the above SINR and rate expressions are understood as instantaneous realizations under a given blockage state. In the numerical evaluation, the performance of EDMA can be further assessed through averaged metrics over multiple blockage realizations, which allows the proposed framework to bridge idealized channel abstraction and more realistic environment-aware deployment analysis. In other words, the random variables $b_{k,n}$ affect the instantaneous effective channels and hence the instantaneous SINR values, while the reported performance trends can be interpreted statistically through averaging over different blockage realizations. Accordingly, users experiencing severe blockage are not assumed to be automatically serviceable under all PA configurations. Their service feasibility depends on whether the selected active PA set can still provide sufficient desired-link support under the imposed quality of service (QoS) constraints.

### 2.2. Problem Formulation

To explore the achievable tradeoff between spectral efficiency (SE) and energy efficiency (EE) in the considered downlink PASS system, we formulate a unified optimization framework based on the achievable-rate expressions derived in the previous subsection. The objective is to characterize the tradeoff between achievable system throughput and transmit energy consumption, while guaranteeing the QoS requirements of all users. Different multiple access mechanisms, namely TDMA and EDMA, are distinguished by their achievable-rate expressions, while sharing the same single-RF-chain power-consumption model introduced in the system model. Let $P_{k}$ denote the transmit power allocated to user *k*, and let $R_{k}^{X}$ denote the achievable downlink rate of user *k* under scheme X∈{TDMA,EDMA}. In this paper, $R_{k}^{X}$ is measured in bit/s/Hz under normalized system bandwidth. Therefore, $R_{k}^{X}$ already represents the spectral-efficiency contribution of user *k*, rather than an unnormalized throughput measured in bit/s. Accordingly, the system sum spectral efficiency is defined as

(15) $${\mathrm{SE}}^{X}≜\sum_{k=1}^{K}R_{k}^{X},\;$$

and the energy efficiency is defined as

(16) $${\mathrm{EE}}^{X}≜\frac{{\mathrm{SE}}^{X}}{P_{\mathrm{tot}}}.$$

The SE metric in (15) captures the overall normalized throughput of the system, whereas (16) measures how efficiently the consumed hardware power is converted into useful information delivery. In general, maximizing SE tends to favor more aggressive transmission and higher radiated power, while maximizing EE prefers a more power-efficient operating point. Therefore, the considered design naturally leads to a tradeoff between these two objectives.

Directly maximizing ${\mathrm{EE}}^{X}$ results in a fractional and highly non-convex optimization problem. To enable tractable analysis and systematic characterization of the SE–EE tradeoff, we adopt a multi-objective optimization perspective that captures the balance between maximizing SE and reducing the total transmit power, while explicitly enforcing QoS constraints.

For the EDMA-enabled PASS considered in this paper, the original design problem can be viewed as a tradeoff-oriented multi-objective optimization problem, in which the system SE is to be improved while the radiated transmit power is to be reduced. Under the normalized-bandwidth formulation, this can be expressed as

(17) $$\begin{array}{cc} \max_{\{P_{k}\},\;z}\; & {\mathrm{SE}}^{X}\left(\left\{P_{k}\right\},z\right),\\ \min_{\{P_{k}\},\;z}\; & \sum_{k=1}^{K}P_{k}. \end{array}$$

where z denotes the PA activation variable over the predefined candidate set along the waveguide. This formulation emphasizes that the system should simultaneously pursue high throughput and low transmit power expenditure.

To this end, we formulate a multi-objective optimization problem (MOOP) that jointly considers transmit power allocation and PA selection. Specifically, the design variables include the transmit powers $P_{k}$ assigned to the users and the PA selection variable z associated with the activated pinching antennas over the predefined candidate set along the waveguide. These variables are coupled in the achievable downlink rates through the effective channels and also affect the resulting system energy consumption.

To obtain a tractable scalar formulation from the MOOP in (17), we adopt an ε-constraint-type reformulation, in which the SE objective is maximized while a lower bound on the SE–power tradeoff is imposed through a design parameter μ≥0. The resulting parameterized problem is written as

(18a) $$\begin{array}{cc} \max_{\{P_{k}\},\;z}\;\; & {\mathrm{SE}}^{X}\left(\left\{P_{k}\right\},z\right) \end{array}$$

(18b) $$\begin{array}{cc} s.t.\;\; & {\mathrm{SE}}^{X}\left(\left\{P_{k}\right\},z\right)-\mu P_{\mathrm{tot}}\left(\left\{P_{k}\right\},z\right)\geq 0, \end{array}$$

(18c) $$\begin{array}{cc} \; & 0\leq P_{k}\leq P_{\max},\;\forall k\in K, \end{array}$$

(18d) $$\begin{array}{cc} \; & R_{k}^{X}\left(\left\{P_{k}\right\},z\right)\geq \gamma_{k},\;\forall k\in K, \end{array}$$

(18e) $$\begin{array}{cc} \; & z\in F_{\mathrm{PA}}, \end{array}$$

where μ is a nonnegative tradeoff parameter. By varying μ, different operating points on the SE–EE tradeoff can be explored.

Here, $P_{\max}$ denotes the maximum allowable transmit power and $\gamma_{k}$ represents the minimum achievable-rate requirement of user *k*, which explicitly enforces per-user QoS guarantees. Constraint (18b) imposes the required SE–power tradeoff level under the adopted parameterization. Constraint (18c) limits the transmit power allocated to each user stream, (18d) guarantees that every user achieves its required service level, and (18e) ensures that the selected PA configuration is physically implementable over the predefined candidate set. Hence, the feasible set simultaneously reflects transmission-power limitations, user-service requirements, and deployment constraints of the PASS architecture.

Since PA locations are selected from a predefined finite set of candidate positions, the deployment design considered in this paper is modeled as a discrete PA selection problem. In addition, the environment-dependent visibility parameters are treated as given statistical characteristics of the propagation scenario, while the optimization determines only the active PA configuration and the transmit power allocation under these visibility conditions.

Although the MOOP in (17) clearly captures the SE–EE tradeoff, it is not directly convenient for algorithm design because SE and EE are intrinsically coupled through both the achievable-rate expressions and the hardware-aware total power-consumption model. Therefore, in the next section, we convert this tradeoff-oriented formulation into a parameterized single-objective problem by means of an ε-constraint reformulation, as shown in (21).

It should be emphasized that, although the above MOOP is formulated over both transmit power and PA selection variables, the resulting non-convex problem is not solved via simultaneous joint updates. Instead, an alternating optimization framework is adopted, in which one variable block is updated while the other is fixed, and the two blocks are optimized sequentially.

It is worth noting that the formulation in (18) applies to both TDMA and EDMA. The two schemes differ only in their achievable-rate functions $R_{k}^{X}\left(\cdot \right)$. TDMA achieves interference-free transmission via time orthogonality, whereas EDMA enables concurrent multi-user transmission under the given environment-dependent visibility structure. In EDMA, the interference pattern is not created by changing the physical environment itself, but by selecting different active PA configurations over the predefined candidate set, which leads to different effective link-utilization patterns under the same statistical blockage conditions. From the mathematical point of view, TDMA can be interpreted as a simplified special case of the same unified framework. Since only one user is served in each time slot, no concurrent inter-user interference is present under TDMA. As a result, the PA-related design under TDMA only affects the desired-link quality of the scheduled user, and the associated optimization problem is substantially simpler than that under EDMA. Therefore, while both TDMA and EDMA are evaluated under the same SE–EE framework and the same hardware-aware power-consumption model, the PA selection and power-allocation problems under TDMA reduce to a simpler baseline design without the need for interference-oriented PA coordination. Therefore, the above formulation provides a unified foundation for characterizing the SE–EE tradeoff and for identifying the operating regimes in which EDMA becomes energy-efficiently preferable to conventional TDMA while satisfying user QoS requirements.

## 3. Optimization and Algorithm Design

In this section, we develop an energy-efficient optimization framework for downlink transmission in PASS under EDMA. Unlike conventional multiple access schemes such as TDMA, which primarily rely on time-domain orthogonality or transmit power allocation to manage multi-user interference, EDMA exploits the structural reconfigurability of PA activation and assignment over a discrete set of feasible deployment points along the waveguide. It should be emphasized that the detailed optimization procedure developed in this section is mainly tailored to EDMA, because concurrent multi-user transmission gives rise to a nontrivial interference-management problem. By contrast, under TDMA, only one user is active in each time slot, and thus, the corresponding PA-related design reduces to a simpler desired-link-oriented baseline problem without explicit interference-suppression stages. This distinction also explains why the TDMA benchmark in the numerical results is evaluated under the same hardware-aware SE–EE framework, while the EDMA case requires the more elaborate PA selection and power-allocation coordination developed below.

To systematically characterize the SE–EE tradeoff under EDMA, we first reformulate the original tradeoff-oriented optimization problem into a parameterized single-objective problem. This reformulation is motivated by the intrinsic coupling between spectral efficiency and hardware-aware power consumption, as well as the non-convexity of the resulting design problem, and enables a tractable exploration of the operating tradeoff between spectral efficiency and energy consumption under explicit QoS constraints. Based on the resulting single-objective formulation, a practical and physically interpretable solution framework is developed, which explicitly reveals how PA activation and power allocation should be coordinated to achieve energy-efficient operation in EDMA-enabled PASS systems.

### 3.1. Parameterized Reformulation via Energy-Efficiency Constraint

Directly maximizing (16) is difficult because the objective is fractional and the achievable-rate terms embedded in ${\mathrm{SE}}^{X}$ are themselves non-concave under EDMA. To expose the SE–EE tradeoff in a more tractable manner, we adopt an ε-constraint-type reformulation. Specifically, a prescribed energy-efficiency threshold μ is introduced and the system is required to operate at an EE level no smaller than this target, i.e.,

(19) $${\mathrm{EE}}^{X}=\frac{{\mathrm{SE}}^{X}\left(\left\{P_{k}\right\},z\right)}{P_{\mathrm{tot}}\left(\left\{P_{k}\right\},z\right)}\geq \mu .$$

Since $P_{\mathrm{tot}}\left(\left\{P_{k}\right\},z\right)>0$ always holds in the considered system, (19) can be equivalently written as

(20) $${\mathrm{SE}}^{X}\left(\left\{P_{k}\right\},z\right)-\mu P_{\mathrm{tot}}\left(\left\{P_{k}\right\},z\right)\geq 0.$$

The parameter μ is not an optimization variable, but a prescribed EE target used to characterize different operating points on the SE–EE tradeoff curve. Under a given μ, maximizing the achievable SE while satisfying (20) leads to the following parameterized single-objective problem:

(21a) $$\begin{array}{cc} \max_{\{P_{k}\},\;z}\;\; & {\mathrm{SE}}^{X}\left(\left\{P_{k}\right\},z\right) \end{array}$$

(21b) $$\begin{array}{cc} s.t.\;\; & {\mathrm{SE}}^{X}\left(\left\{P_{k}\right\},z\right)-\mu P_{\mathrm{tot}}\left(\left\{P_{k}\right\},z\right)\geq 0, \end{array}$$

(21c) $$\begin{array}{cc} \; & 0\leq P_{k}\leq P_{\max},\;\forall k\in K, \end{array}$$

(21d) $$\begin{array}{cc} \; & R_{k}^{X}\left(\left\{P_{k}\right\},z\right)\geq \gamma_{k},\;\forall k\in K, \end{array}$$

(21e) $$\begin{array}{cc} \; & z\in F_{\mathrm{PA}}. \end{array}$$

Problem (21) preserves the essential tradeoff structure of the original formulation while being more suitable for algorithm design, because the target EE level is now explicitly controlled through the parameter μ. By varying μ, one can explore different feasible operating points and thereby characterize the SE–EE tradeoff of the considered EDMA-enabled PASS system.

### 3.2. Alternating Optimization Structure

The optimization variables in (21) naturally fall into two categories: the transmit power vector $\left\{P_{k}\right\}$ and the discrete PA activation/assignment variables represented by z. In EDMA-enabled PASS, these two variable blocks play fundamentally different roles. Specifically, the statistical visibility and blockage characteristics of wireless links are determined by the propagation environment, whereas the selected candidate PAs determine which of these candidate PA–user links are effectively utilized for transmission under the given visibility conditions. In particular, z affects the effective channels through PA selection under environment-dependent probabilistic link visibility, while $\left\{P_{k}\right\}$ adjusts the operating point on the SE–EE tradeoff under the interference pattern induced by the selected active PAs.

Even after the above reformulation, the resulting optimization problem remains highly non-convex for multiple reasons. First, under EDMA the achievable-rate terms $R_{k}^{X}$ are generally non-concave in the transmit powers because the interference terms in the denominators of the SINRs couple the power variables across different users. Second, the QoS constraints $R_{k}^{X}\left(\left\{P_{k}\right\},z\right)\geq \gamma_{k}$ are therefore non-convex as well. Third, the PA selection variable z is discrete and combinatorial, which introduces a large-scale non-convex feasible set. Fourth, the effective channels depend on the selected active PAs, which further couples the continuous power variables and the discrete PA variables.

Due to this coupling, the resulting optimization problem over $\left\{P_{k}\right\}$ and z is highly non-convex. The non-convexity arises because both the achievable-rate expressions and the EE constraint in (21b) depend on $\left\{P_{k}\right\}$ and z in a coupled manner through the effective channels and the resulting interference terms. To achieve a balance between performance and computational tractability, we adopt an alternating optimization (AO) strategy. PA activation selects transmission links over the candidate set under the probabilistic visibility structure, thereby affecting the effective interference topology experienced by the users. It should also be emphasized that the problem is not solved via simultaneous joint updates. Instead, one variable block is optimized while the other is fixed, and the two blocks are updated sequentially. The key motivation is that, when one variable block is fixed, the resulting subproblem admits a clearer structure and becomes more amenable to efficient optimization. Accordingly, the AO framework alternates between optimizing the continuous transmit power variables and optimizing the discrete PA selection variables until convergence. More specifically, when z is fixed, the remaining subproblem reduces to a continuous power-allocation problem under QoS and EE constraints; when $\left\{P_{k}\right\}$ is fixed, the remaining subproblem reduces to a discrete PA selection problem over the predefined candidate set. This two-block decomposition provides the basis for the AO algorithm developed in the following subsections.

### 3.3. Power Optimization with Fixed Pinching-Antenna Activation

For a given PA activation pattern z, the equivalent channel coefficients $H_{k,i}\left(z\right)$ are determined by the selected candidate PAs and the corresponding probabilistic visibility conditions. Accordingly, the SINR and the achievable rate of each user under EDMA follow directly from (13) and (14), with the dependence on z explicitly captured through $H_{k,i}\left(z\right)$.

To obtain a tractable power-allocation design under probabilistic blockage, we adopt an expected-rate formulation, in which the utility and QoS constraints are evaluated in a statistical sense with respect to the visibility model introduced in Section 2. For a fixed z and a given EE tradeoff parameter μ, we adopt the following subtractive utility function

(22) $$U\left(\left\{P_{k}\right\}|z,\mu \right)≜\sum_{k=1}^{K}{\bar{R}}_{k}^{\mathrm{EDMA}}-\mu \left(\frac{1}{\xi_{\mathrm{pa}}}\sum_{k=1}^{K}P_{k}+P_{\mathrm{RF}}+N_{\mathrm{act}}P_{\mathrm{PA}}+P_{\mathrm{sta}}\right),$$

which is induced by the EE-aware reformulation introduced in the previous subsection. Here, μ is not an arbitrary weighting factor, but a prescribed EE-related parameter with unit bit/J/Hz. Therefore, the term $\mu P_{\mathrm{tot}}$ has unit bit/s/Hz, which is dimensionally consistent with the expected-rate term. As a result, (22) is not a direct combination of two objectives with mismatched units, but a scalarized and dimensionally consistent utility function that balances throughput and power expenditure under the prescribed EE target.

By substituting the EDMA achievable-rate expressions into the EE-aware utility, the power optimization subproblem for a fixed z can be formulated as.

(23a) $$\begin{array}{cc} \max_{\left\{P_{k}\right\}}\;\; & \sum_{k=1}^{K}{\bar{R}}_{k}^{\mathrm{EDMA}}-\mu \left(\frac{1}{\xi_{\mathrm{pa}}}\sum_{k=1}^{K}P_{k}+P_{\mathrm{RF}}+N_{\mathrm{act}}P_{\mathrm{PA}}+P_{\mathrm{sta}}\right) \end{array}$$

(23b) $$\begin{array}{cc} s.t.\;\; & {\bar{R}}_{k}^{\mathrm{EDMA}}\geq \gamma_{k},\;\forall k\in K, \end{array}$$

(23c) $$\begin{array}{cc} \; & 0\leq P_{k}\leq P_{\max},\;\forall k\in K, \end{array}$$

where ${\bar{R}}_{k}^{\mathrm{EDMA}}$ denotes the expected achievable downlink rate of user *k* under EDMA, evaluated with respect to the probabilistic visibility model introduced in Section 2. The objective in (23a) aims to maximize the expected sum spectral efficiency while penalizing excessive power expenditure according to the prescribed EE-related parameter μ. Constraint (23b) ensures that each user satisfies its minimum service requirement in the expected-rate sense, and (23c) restricts the feasible transmit power range of each user stream.

Due to the coupling among users through multi-user interference, the objective function and the QoS constraints in (23) are non-convex with respect to the transmit power variables $P_{k}$. More specifically, the non-convexity arises because each expected-rate term depends on an SINR expression whose denominator contains interference contributions from other users’ transmit powers. Therefore, both the utility in (23a) and the QoS constraints in (23b) involve coupled non-convex functions of $P_{k}$.

To address this challenge, we employ the successive convex approximation (SCA) technique. At each iteration, the non-convex rate expressions induced by the interference coupling are locally approximated by convex surrogate functions constructed via first-order Taylor expansion around the current operating point. This procedure yields a sequence of convex subproblems with respect to $\left\{P_{k}\right\}$, each of which can be efficiently solved using standard convex optimization tools.

Under standard assumptions commonly adopted in SCA-based methods, the proposed iterative procedure guarantees a non-decreasing objective value and convergence to a stationary point satisfying the Karush–Kuhn–Tucker (KKT) conditions of the original power optimization subproblem.

### 3.4. Pinching-Antenna Selection Optimization with Fixed Power Allocation

With the transmit power vector $\left\{P_{k}\right\}$ fixed, the system performance depends on the selected active candidate PAs through the equivalent channel gains $H_{k,i}\left(z\right)$. In this case, the PA selection optimization serves as a surrogate design step that indirectly improves the original SE–EE objective by modifying the effective link-utilization pattern and the resulting interference structure under the given probabilistic visibility conditions. Expanding the channel expression yields

(24) $$|H_{k,i}{\left(z\right)|}^{2}={\left|\sum_{n\in A_{i}}b_{k,n}h_{k,n}\right|}^{2},$$

where $b_{k,n}$ is the Bernoulli visibility variable introduced in Section 2 and the active set $A_{i}$ is determined from the candidate set according to z.

Directly optimizing the discrete PA activation variables with respect to the original SE–EE objective is combinatorial and highly challenging due to the non-convex and geometry-dependent nature of $H_{k,i}\left(z\right)$. Therefore, instead of directly solving the original discrete SE–EE problem, we adopt a low-complexity surrogate-based PA selection strategy. The key idea is to use physically meaningful metrics that capture the two dominant effects of PA activation under fixed power allocation, namely, inter-user interference and desired-link strength. To exploit the environment-dependent interference characteristics of EDMA while maintaining computational tractability, the PA selection optimization is carried out using a structured two-stage heuristic strategy. Here, the corresponding interference behavior is jointly determined by the given environment-dependent visibility structure and by which candidate PAs are selected, rather than by actively modifying the environment itself. The rationale behind the two-stage design is as follows. Under EDMA, severe inter-user coupling may significantly degrade the achievable rates of all users, even when the desired links are moderately strong. Hence, the first priority is to reduce harmful interference. However, an overly aggressive interference-oriented selection may also discard useful desired links, especially for users experiencing unfavorable blockage conditions. Therefore, after the interference level is sufficiently reduced, a second refinement stage is introduced to reinforce the desired signals without reintroducing excessive interference. As a result, the proposed PA selection method does not attempt to directly maximize the original SE–EE objective. Instead, it sequentially optimizes two surrogate metrics that are closely related to the final system performance, thereby providing a practical balance between algorithmic tractability and physical interpretability. More precisely, PA activation determines which candidate links are utilized under the given blockage statistics, so the design must balance interference suppression and desired-link preservation, especially for users that may experience severe blockage.

#### 3.4.1. Interference Suppression Stage

In the first stage, the primary objective is to reduce multi-user interference by exploiting the given environment-dependent visibility structure. Specifically, the aggregate interference power is quantified as

(25) $$J_{\mathrm{int}}\left(z\right)=\sum_{k=1}^{K}\sum_{i\neq k}P_{i}\;E\;[|H_{k,i}{\left(z\right)|}^{2}].$$

This metric captures the overall strength of non-intended links under the fixed power allocation. A smaller value of $J_{\mathrm{int}}\left(z\right)$ indicates weaker average cross-user coupling and, hence, a more favorable interference topology for concurrent EDMA transmission. Therefore, the first-stage PA update aims to find a candidate PA configuration that reduces $J_{\mathrm{int}}\left(z\right)$ as much as possible over the feasible search neighborhood. By adjusting the PA activation pattern over the predefined candidate set, the system changes which statistically visible links contribute to interference, thereby altering the interference topology in a statistical environment-aware sense.

The interference suppression stage aims to establish a favorable interference structure before enhancing desired links, thereby preventing excessive performance degradation caused by strong inter-user coupling.

#### 3.4.2. Desired-Signal Enhancement Stage

After sufficient interference suppression is achieved, the active PA set is further refined to enhance the desired signal strength. The aggregate desired-signal power is measured by

(26) $$J_{\mathrm{sig}}\left(z\right)=\sum_{k=1}^{K}P_{k}\;E\;[|H_{k,k}{\left(z\right)|}^{2}].$$

Maximizing $J_{\mathrm{sig}}\left(z\right)$ improves the effective channel gains of intended links, thereby compensating for potential signal attenuation introduced by aggressive interference suppression in the previous stage. In other words, once the dominant interference has been suppressed, the second stage seeks to recover or enhance useful desired-link power so that the resulting PA configuration is not overly conservative from the viewpoint of throughput. This refinement is particularly important for blocked or weak users, whose intended links may otherwise be excessively degraded by an interference-only criterion. This sequential refinement implicitly balances interference suppression and signal enhancement without introducing additional weighting parameters.

To limit computational complexity and ensure practical deployability, a discrete local search strategy is adopted for PA selection updates. At each iteration, one or several candidate PAs are activated, deactivated, or swapped within the feasible candidate set, and the update is accepted only if it improves the corresponding surrogate objective while satisfying the deployment and cardinality constraints. More specifically, in the first stage, a candidate move is accepted if it decreases $J_{\mathrm{int}}\left(z\right)$ over the current PA neighborhood. In the second stage, the search is continued from the interference-suppressed PA configuration, and a candidate move is accepted if it improves $J_{\mathrm{sig}}\left(z\right)$ without violating the feasibility constraints. Since each accepted update monotonically improves the corresponding surrogate objective, the resulting discrete local-search procedure converges to a stable PA configuration after a finite number of updates. Within the alternating optimization framework, this procedure yields a stable PA selection update for the fixed power-allocation block and contributes to the overall convergence of the proposed AO algorithm, although global optimality is not claimed. The overall AO-based energy-aware EDMA design procedure is summarized in Algorithm 1.
**Algorithm 1:** Energy-aware EDMA design for downlink PASS1:**Input:** EE-related parameter μ, QoS requirements $\left\{\gamma_{k}\right\}$, maximum transmit power $P_{\max}$, candidate PA set N, feasible PA activation set $F_{\mathrm{PA}}$, convergence tolerance ε.2:**Initialization:** Initialize a feasible PA activation vector $z^{\left(0\right)}\in F_{\mathrm{PA}}$; initialize transmit-power vector $p^{\left(0\right)}=\left\{P_{k}^{\left(0\right)}\right\}$; set iteration index t=0.3: t←t+14: **Power Allocation Update (given $z^{\left(t-1\right)}$):**5: Solve the power-allocation subproblem in (23), i.e.,$$\max_{\left\{P_{k}\right\}}\;\sum_{k=1}^{K}{\bar{R}}_{k}^{\mathrm{EDMA}}-\mu \left(\frac{1}{\xi_{\mathrm{pa}}}\sum_{k=1}^{K}P_{k}+P_{\mathrm{RF}}+N_{\mathrm{act}}P_{\mathrm{PA}}+P_{\mathrm{sta}}\right)$$
subject to$${\bar{R}}_{k}^{\mathrm{EDMA}}\geq \gamma_{k},\;\forall k,\;0\leq P_{k}\leq P_{\max},\;\forall k.$$6: Obtain the updated power vector $p^{\left(t\right)}$.7: **PA Selection Update (given $p^{\left(t\right)}$):**8: **Stage 1: Interference Suppression**9: Starting from $z^{\left(t-1\right)}$, perform discrete local search over $F_{\mathrm{PA}}$ and accept a feasible PA update if it decreases$$J_{\mathrm{int}}\left(z\right)=\sum_{k=1}^{K}\sum_{i\neq k}P_{i}^{\left(t\right)}\;E\;[|H_{k,i}{\left(z\right)|}^{2}].$$10: Denote the resulting interference-suppressed PA configuration by ${\tilde{z}}^{\left(t\right)}$.11: **Stage 2: Desired-Signal Enhancement**12: Starting from ${\tilde{z}}^{\left(t\right)}$, continue discrete local search over $F_{\mathrm{PA}}$ and accept a feasible PA update if it improves$$J_{\mathrm{sig}}\left(z\right)=\sum_{k=1}^{K}P_{k}^{\left(t\right)}\;E\;[|H_{k,k}{\left(z\right)|}^{2}].$$13: Set the refined PA activation vector as $z^{\left(t\right)}$.14: Compute the overall utility value
$$U^{\left(t\right)}=\sum_{k=1}^{K}{\bar{R}}_{k}^{\mathrm{EDMA}}-\mu \left(\frac{1}{\xi_{\mathrm{pa}}}\sum_{k=1}^{K}P_{k}^{\left(t\right)}+P_{\mathrm{RF}}+N_{\mathrm{act}}P_{\mathrm{PA}}+P_{\mathrm{sta}}\right).$$$\;\;\;\;\;\;\;\;|U^{\left(t\right)}-U^{\left(t-1\right)}|\leq \varepsilon$15:**Output:** Optimized transmit-power vector $p^{★}$, optimized PA activation vector $z^{★}$, and the corresponding performance pair $\left({\mathrm{SE}}^{\mathrm{EDMA}},{\mathrm{EE}}^{\mathrm{EDMA}}\right)$.

## 4. Numerical Results

In this section, numerical results are presented to evaluate the SE–EE tradeoff of the considered pinching-antenna-assisted multiple access schemes. The performance of the proposed EDMA framework is compared with conventional TDMA and a fixed pinching-antenna baseline under different transmit power levels. Unless otherwise specified, the simulation parameters are set as follows. The BS employs a single dielectric waveguide deployed at a height of d=3 m above the ground. The main simulations consider K=5 single-antenna users in a service region with lateral width D=10 m. Along the waveguide direction, the default deployment extent is set to $D_{\mathrm{leng}}=2D=20$ m, unless otherwise specified. To reflect practical discrete PASS deployment, the waveguide is partitioned into *K* user-associated segments, each containing $N_{c}=9$ candidate PA positions. Hence, the total number of candidate PA points is $N=KN_{c}=45$. In the default setting, one PA is selected from each segment, so that the number of activated PAs is $N_{\mathrm{act}}=K=5$. All users are randomly distributed in the indoor communication region, with each user constrained to lie in its corresponding predefined segment. The carrier frequency is set to $f_{c}=28$ GHz, corresponding to the free-space wavelength $\lambda =c/f_{c}$. The transmit power is swept from −10 dBm to 30 dBm, and the noise power is fixed to $\sigma^{2}=10^{-12}$ W. For the hardware-aware power model, the power-amplifier efficiency is set to $\xi_{\mathrm{pa}}=0.35$, the RF-chain power is $P_{\mathrm{RF}}=0.60$ W, the per-active-PA control power is $P_{\mathrm{PA}}=0.03$ W, and the static circuit power is $P_{\mathrm{sta}}=0.40$ W. The nominal interference parameter is set to ϕ=0.02, where ϕ is the decay parameter in the probabilistic neighbor-interference model.

For TDMA, users are served orthogonally with equal time allocation, and the achievable spectral efficiency is scaled by 1/K to account for time sharing. For EDMA-based schemes, users are served concurrently, and mutual interference is modeled through the same probabilistic neighbor-interference model. For a fair comparison, this model is also applied to the fixed pinching-antenna baseline; the difference is that the PA locations are fixed in the baseline, whereas they are optimized in EDMA. For the proposed AO-based EDMA design, the maximum number of outer iterations is set to 12, the maximum number of local PA-search passes is set to 4, and the stopping tolerance is $10^{-4}$. All numerical results are averaged over a sufficiently large number of independent Monte Carlo realizations of user locations, ensuring statistically meaningful performance evaluation under different multiple access strategies.

**Figure 2:**
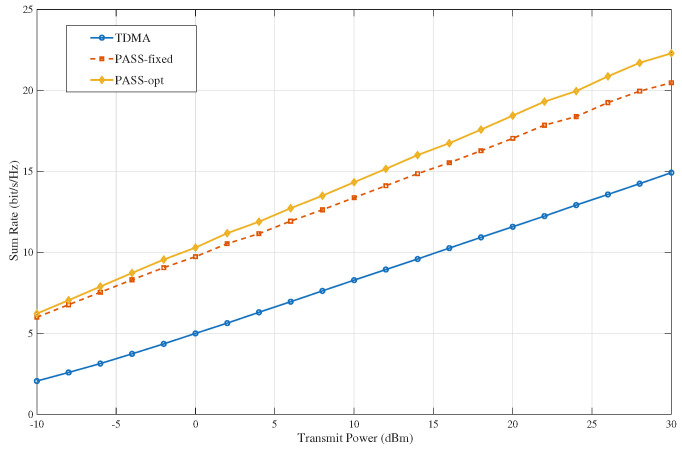
Sum-rate performance versus transmit power for different multiple access schemes.

Figure 2 illustrates the achievable sum rate versus transmit power for different multiple access schemes with K=5 users. PASS-based EDMA consistently outperforms TDMA across the entire power range. The main reason is that TDMA suppresses interference through time orthogonalization, but this also limits each user to only a fraction of the transmission time. In contrast, EDMA supports concurrent transmission and exploits the environment-dependent visibility differences among candidate pinching antennas, thereby achieving more efficient spatial reuse. The figure also shows that optimizing the PA locations provides an additional gain over the fixed PASS deployment. This is because PA selection not only enhances desired links, but also changes which statistically visible links are utilized under the given blockage conditions, leading to a more favorable interference structure. Moreover, the gain of PA optimization becomes more evident at medium-to-high transmit power, where the system gradually shifts from being noise-limited to interference-limited. In this regime, interference-aware PA selection plays a more important role in improving the sum-rate performance.

**Figure 3:**
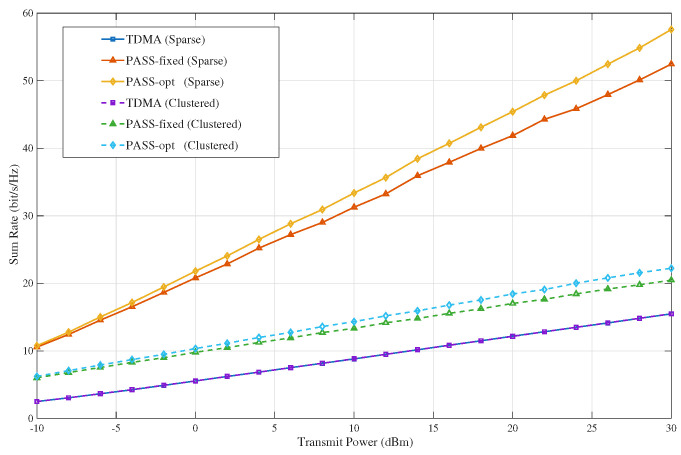
Sum-rate versus transmit power under sparse and clustered user distributions along the waveguide.

Figure 3 illustrates the achievable sum rate versus transmit power under two different spatial user distributions along the waveguide direction, namely the Sparse case and the Clustered case, with K=5 users. The TDMA baseline is nearly unaffected by the user distribution, because users are served orthogonally in time and the performance mainly depends on the single-user link quality in each slot. In contrast, PASS-based EDMA is highly sensitive to the user distribution. When users are sparsely distributed, inter-user coupling is weaker and the resulting interference geometry is more favorable for concurrent transmission. When users are clustered, the overlap among effective links becomes stronger, which intensifies interference and reduces the achievable sum rate. Figure 3 also shows that optimizing the pinching-antenna locations provides additional gains in both cases. The reason is that PA selection can better adapt the effective link-utilization pattern to the given user geometry and blockage conditions, thereby improving the balance between desired-signal enhancement and interference suppression. This gain becomes more visible at higher transmit power, where the system gradually shifts toward an interference-limited regime.

**Figure 4:**
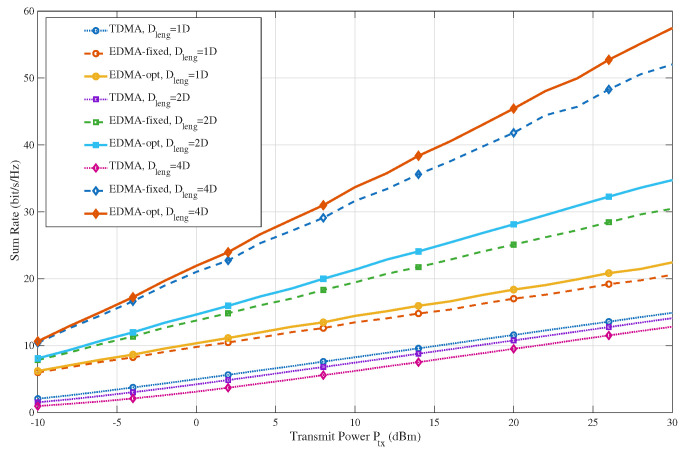
Sum-rate versus transmit power under different deployment extents along the waveguide ($D_{\mathrm{leng}}\in \left\{1D,2D,4D\right\}$).

Figure 4 compares the sum-rate performance under different deployment extents along the waveguide, i.e., $D_{\mathrm{leng}}\in \left\{1D,2D,4D\right\}$. A larger $D_{\mathrm{leng}}$ corresponds to a more spatially spread user distribution and therefore weaker neighbor interference in EDMA. The figure shows opposite trends for TDMA and PASS-based EDMA. As $D_{\mathrm{leng}}$ increases, TDMA gradually degrades because the average user distance to the BS becomes larger, which weakens the desired-link strength. In contrast, EDMA benefits significantly from a larger deployment extent, since wider spatial separation reduces inter-user coupling and creates a more favorable interference geometry for concurrent transmission. Figure 4 also shows that optimizing the pinching-antenna locations consistently improves the EDMA performance for all deployment extents. The reason is that PA selection can further adapt the effective link-utilization pattern to the given user geometry, thereby improving the balance between desired-signal enhancement and interference suppression. Moreover, this optimization gain becomes more visible when $D_{\mathrm{leng}}$ is larger or when the transmit power is higher, because in these cases, the system relies more heavily on interference-aware spatial coordination rather than purely on link strength.

**Figure 5:**
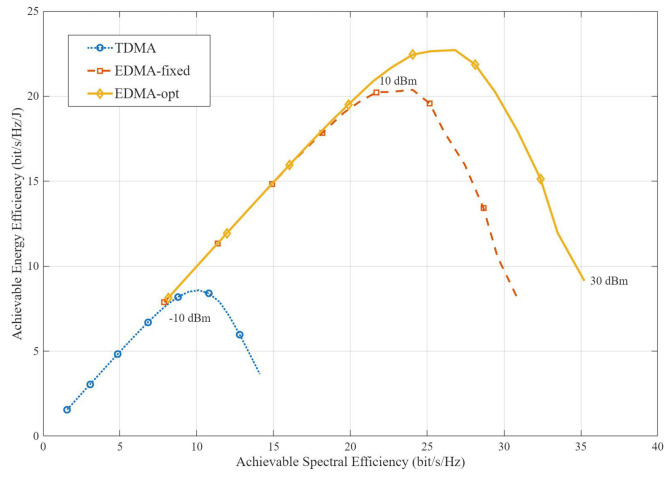
Achievable SE–EE tradeoff of TDMA and EDMA schemes.

Figure 5 illustrates the achievable SE–EE tradeoff for TDMA, EDMA-fixed, and EDMA-opt under K=5 and $D_{\mathrm{leng}}=2D$. All schemes exhibit the typical rise-and-fall EE trend as SE increases. This is because at low-to-medium transmit power, the SE gain dominates the increase in power consumption, whereas at high transmit power, the marginal SE improvement becomes smaller and the radiated-power cost becomes dominant. A more important observation is that the EDMA schemes achieve a substantially enlarged SE–EE region compared with TDMA. The reason is that TDMA avoids interference through time orthogonalization but sacrifices spectral efficiency, while EDMA supports concurrent multi-user transmission and can better exploit the spatial degrees of freedom provided by PASS. As a result, EDMA converts transmit power into useful SE gains over a much wider operating range. Figure 5 also shows that pinching-antenna optimization further improves the SE–EE frontier over the fixed PASS baseline, especially in the medium-to-high SE regime. This is because PA optimization better adapts the effective link-utilization pattern to the given blockage and interference conditions, thereby improving the balance between desired-signal enhancement and interference suppression.

**Figure 6:**
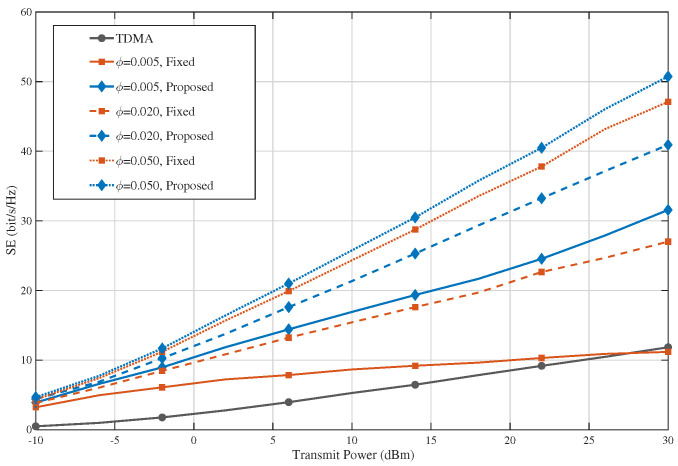
Impact of the blockage parameter on spectral efficiency.

Figure 6 shows the spectral-efficiency performance of TDMA, the fixed PA baseline, and the proposed AO-based design under different blockage parameters. In the adopted model, the blockage parameter only affects the visibility of interference links in EDMA, and therefore, the TDMA baseline remains unchanged with respect to this parameter. It can be observed that the proposed method consistently achieves the highest SE across the entire transmit power range, while the fixed design also outperforms TDMA in most cases. In addition, as the blockage parameter increases, the SE of both the fixed and the proposed EDMA schemes improves significantly. This is because a larger blockage parameter corresponds to faster decay of interference-link visibility, which weakens the effective inter-user interference and makes the environment-driven multiple access mechanism more beneficial. These results demonstrate that the proposed scheme maintains stable SE gains under different blockage conditions and that its advantage becomes more pronounced when the environment is more favorable for interference isolation.

**Figure 7:**
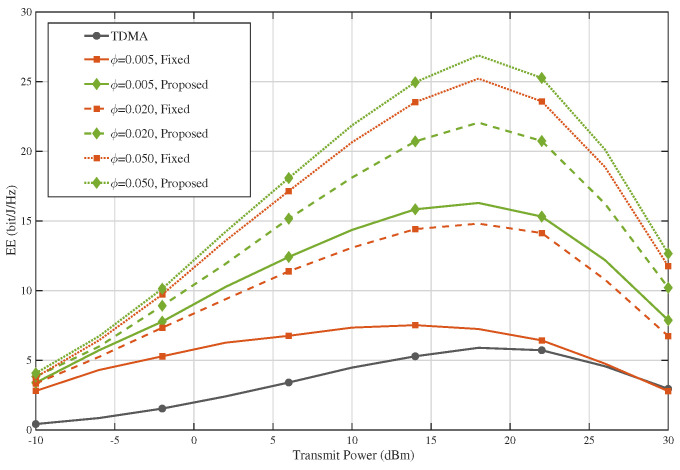
Impact of the blockage parameter on energy efficiency.

Figure 7 presents the corresponding energy-efficiency results under different blockage parameters. Unlike the SE curves, the EE curves exhibit a clear non-monotonic behavior with respect to transmit power, first increasing and then decreasing. This trend is expected because, in the low-to-medium power regime, the achievable rate increases faster than the total power consumption, whereas in the high-power regime, the marginal rate gain diminishes while the energy cost continues to grow. Among all schemes, the proposed design consistently achieves the highest EE under all blockage settings, and its advantage over both TDMA and the fixed baseline is especially evident in the medium-power region where the EE peaks occur. Similar to the SE results, a larger blockage parameter leads to higher EE for the EDMA-based schemes, since stronger interference suppression enables more efficient use of the transmitted energy. Therefore, the proposed method not only provides superior spectral efficiency but also preserves clear energy-efficiency gains across different blockage environments, confirming its robustness from both the SE and EE perspectives.

**Figure 8:**
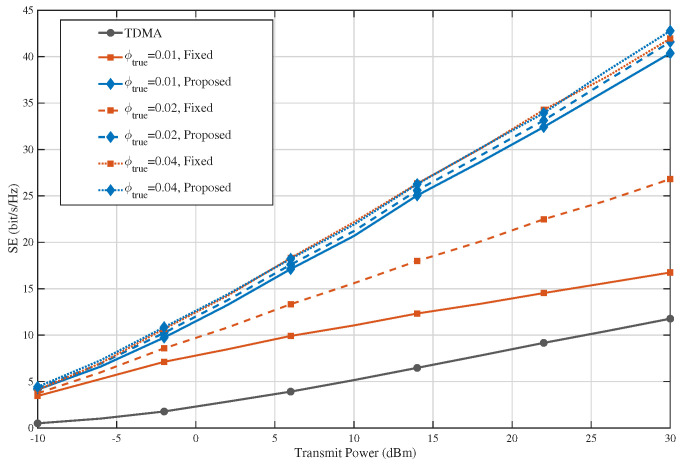
SE under blockage-model mismatch.

Figure 8 evaluates the spectral-efficiency performance under blockage-model mismatch, where the proposed PA selection and power allocation are optimized using a fixed design parameter $\phi_{\mathrm{design}}=0.02$, while the actual performance is re-evaluated under different true blockage parameters $\phi_{\mathrm{true}}\in \left\{0.01,0.02,0.04\right\}$. It can be observed that the proposed method consistently outperforms both the TDMA baseline and the fixed-PA baseline over the entire transmit power range, even when $\phi_{\mathrm{true}}\neq \phi_{\mathrm{design}}$. Although the absolute SE varies with the true blockage condition, the overall superiority of the proposed design remains stable. In particular, a larger $\phi_{\mathrm{true}}$ leads to stronger suppression of interference-link visibility, which improves the SE of the EDMA-based schemes. More importantly, the performance ranking among the compared methods is preserved under all tested mismatch settings. These results indicate that the SE gains of the proposed method are robust to blockage-model errors and do not rely on an exact match between the design model and the true environment.

**Figure 9:**
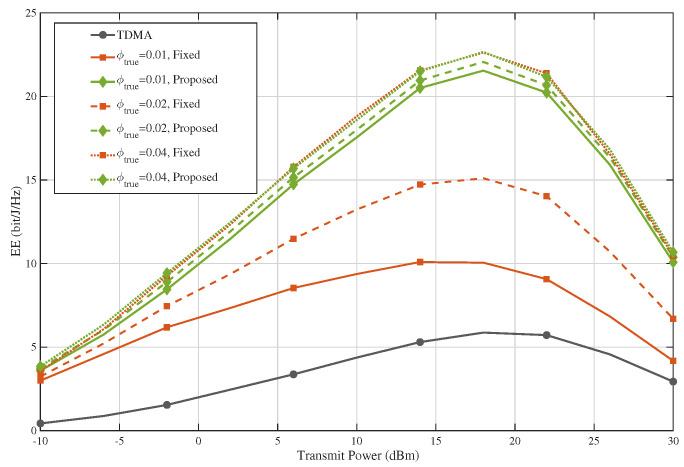
EE under blockage-model mismatch.

Figure 9 presents the corresponding energy-efficiency results under the same blockage-model mismatch setting. Similar to the SE case, the proposed method maintains clear EE gains over TDMA and the fixed-PA baseline for all tested values of $\phi_{\mathrm{true}}$. The EE curves exhibit the expected non-monotonic behavior with respect to transmit power, first increasing and then decreasing, since the rate improvement dominates in the low-to-medium power regime while the power consumption becomes dominant at high transmit power. Under different true blockage conditions, the absolute EE values change to some extent, but the proposed method consistently remains the best-performing scheme. This shows that the energy-efficiency advantage of the proposed design is not sensitive to moderate mismatch in the blockage parameter. Therefore, Figure 6 and Figure 7 together demonstrate that the main SE/EE conclusions of the paper remain valid even when the blockage model used for design is not perfectly matched to the actual propagation condition.

## 5. Conclusions

This paper studied the SE–EE tradeoff of downlink PASS under EDMA. By exploiting the reconfigurable radiation geometry of pinching antennas, EDMA enables concurrent multi-user transmission through environment-dependent interference patterns, rather than conventional time-domain orthogonalization. Within a single-waveguide, single-RF-chain PASS architecture, a unified framework was developed to characterize and compare the SE–EE behaviors of EDMA and the TDMA baseline. The SE–EE tradeoff was formulated as a coupled optimization problem involving transmit power allocation and pinching-antenna selection over a predefined candidate set. Using an ϵ-constraint reformulation and an alternating optimization strategy, an energy-aware EDMA design was obtained that explicitly captures the coupling between power control and the resulting interference structure under the given blockage conditions. The results show that EDMA fundamentally alters the SE–EE operating regime and can achieve higher energy efficiency than TDMA under a wide range of system and environmental conditions.

The current study is developed under a single-waveguide, single-RF-chain PASS/EDMA architecture and mainly focuses on quasi-static scenarios, where user locations and blockage conditions remain relatively stable within each optimization interval. As a result, the present framework does not explicitly account for online reconfiguration overhead, feedback delay, or rapid environmental variations. In addition, extensions to multi-UAV or multi-waveguide systems are beyond the scope of this work, since such settings would introduce new challenges such as inter-platform coordination, cross-waveguide resource allocation, and distributed interference management. Moreover, although the proposed design is supported by extensive numerical results, experimental validation with measured data and hardware implementation has not yet been included. These limitations suggest several important directions for future work, including low-complexity online reconfiguration in dynamic environments, extensions to multi-platform and multi-waveguide deployments, and the development of a small-scale hardware prototype for experimental verification.

## Data Availability

The original contributions presented in this study are included in the article. Further inquiries can be directed to the corresponding author.

## Nomenclature

K	User index set
*K*	Number of users
$u_{k}$	Position of user *k*
$r_{k,n}$	Distance between user *k* and PA *n*
$h_{k,n}$	PA–user channel coefficient
$b_{k,n}$	Visibility indicator
$A_{i}$	Active PA set for user *i*
$P_{k}$	Transmit power for user *k*
$y_{k}$	Received signal at user *k*
$R_{k}^{X}$	Achievable rate of user *k*
${\mathrm{SE}}^{X}$	System spectral efficiency
$P_{\mathrm{tot}}$	Total power consumption
z	PA activation vector
$\gamma_{k}$	QoS requirement of user *k*
$J_{\mathrm{int}}\left(z\right)$	Aggregate interference metric
N	Candidate PA index set
*N*	Number of candidate PAs
$\psi_{n}$	Position of candidate PA *n*
$\lambda ,\lambda_{g}$	Carrier/guided wavelength
${\tilde{h}}_{k,n}$	Effective channel with blockage
$\rho_{k,n}$	Visibility probability
$H_{k,i}$	Effective channel from stream *i* to user *k*
$P_{\mathrm{tx}}$	Total transmit power
$\sigma^{2}$	Noise power
${\mathrm{SINR}}_{k}^{\mathrm{EDMA}}$	EDMA SINR of user *k*
${\mathrm{EE}}^{X}$	System energy efficiency
$\xi_{\mathrm{pa}}$	Power-amplifier efficiency
$N_{\mathrm{act}}$	Number of activated PAs
$P_{\max}$	Maximum transmit power
$J_{\mathrm{sig}}\left(z\right)$	Aggregate desired-signal metric
